# Annotations

**Published:** 1910-11-26

**Authors:** 


### Annotations.

### The Wandsworth Inquest.

Any politician or social economist who is still unconvinced of the necessity—indeed, the urgency—of legislation for the regulation of the practice of anæsthetics by those without adequate training and qualifications may be recommended to study carefully the report of the inquest held on November 17 in respect of a fatality in the dental chair. The facts elicited were so conclusive that it is hard to understand why the jury refrained from bringing in a verdict of manslaughter instead of merely censuring the operator. What happened can be recounted very briefly. An unregistered dentist conducted a dental clinic under a style which enabled him to defy the letter of the law, while violating its spirit completely. He has been in the habit of administering gas and performing extraction unaided, save by a woman, who takes upon herself the appellation of a nurse, despite the fact that she has not been trained as one. Indeed, the man says he has performed this dual *rôle* thirteen or fourteen hundred times. One day last week he extracted five or six teeth from a man in this manner; shortly after the close of the proceedings the nurse discovered that respiration had ceased, and life was in fact extinct, notwithstanding artificial respiration and other efforts to resuscitate the patient. At autopsy it was found that the patient was suffering from some cardiac condition undiagnosed before the anæsthesia. The most important features of this sad case are these: The administration of a general anæsthetic by the person about to operate, a common practice, but one in the highest degree reprehensible in the patient's interests. The administration of a general anæsthetic by a person who under any scheme of legal regulation would doubtless be prohibited from so doing altogether; the absence of any trained or skilled help; and, finally, a fatality which, in all the circumstances, can confidently be pronounced to have been an avoidable one.

### The Nobel Prize in Medicine.

The Nobel Prize in Medicine has been awarded to Dr. Albrecht Kossel, Professor of Physiology at Heidelberg, one of the best known of German scientists. Born at Rostock, in the Grand Duchy of Mecklenburg-Schwerin, he was educated at that town and at Strassburg, where in 1877 he became assistant to Hoppe-Seyler. In 1883 he was called upon to direct the chemical section of the Physiological Institute at Berlin. Since then he has occupied successively the Chair of Physiology at Marburg and at Heidelberg, where he now resides. In the scientific world the Professor is well known for his microscopical researches into the nature of the human cells and tissues and for his work on the albumins. By awarding him the prize, the Stockholm Institute has given due recognition to a career devoted to difficult and disinterested scientific investigation. This year the prize is of the approximate value of £7,734.

### The American Aviation Accident.

It is satisfactory to note that skilled inquiry, so far as that is possible, has been made into the causes of accidents to aeroplanists. Unhappily, as these are often quickly fatal, the surgery of this subject does not seem a hopeful field of study. Nevertheless one particular suggests itself in which a medical man of high moral and physical efficiency might do good service—namely in helping to keep spectators back from the necessarily unforeseen site of a mishap. The crowd that collects around a hurt man will obey a doctor when it will obey no one else—that is if the doctor knows enough of mob psychology and is strong enough to take charge of the situation, adopting the firmest of tones from the outset. The scene reported by Reuter's cable from Denver was profoundly disgraceful. Mr. Kipling, whose mind seems to be taken up with a vision of the whole animate creation bowing down to the Anglo-Saxon, has described certain Asiatics recently conquered by America as "half-devil and half-child." Yet, if ever a description fitted, "half-devil and half-child" fits those morbid persons of both sexes who, running mad like cattle at the sight of blood, were fighting for souvenirs almost as soon as man and machine had struck the ground. One "rushed to the wreck, pulled out a wooden stay piercing the body, and carried off his trophy dripping with the aviator's blood." Others fought for the gloves on the dead man's hands. How magnificently old Ambroise Paré could have rebuked these little-brained, evil-minded, erethistic degenerates! How speedily—the anachronism may pass—a United Hospital Rugby football team would have enforced his stern commands!

### "The Child."

The first number of this new monthly, which is under the editorship of Dr. Kelynack, has just been issued. We confess to a feeling of disappointment on perusing it. With such a title and such material, both in contributors and possible contributions, it was only excusable to anticipate a magazine which should be really stimulating, and should deal with the manifold problems of child life in an outspoken and frank fashion. Dr. Kelynack has, we are convinced, the courage and the ability to make his new journal not merely what it is bound to be—a popular success, but an effective weapon in the struggle for the recognition of children's interests and the claims of the growing generation upon the public. But there is little evidence of this intention here. The magazine is beautifully printed and excellently illustrated; one or two articles are good, but the mass of the work is scrappy and unsatisfying. The final part of the journal is good for reference purposes, since it gives details of all the various children's organisations. We regret that we have not been able to welcome the new magazine more cordially; it supplies a want, and there is undoubted scope for it, but it should strive to be a really effective organ, and not a mere popular arm-chair periodical of the type now unfortunately so common.

